# Patterns of control beliefs in chronic fatigue syndrome: results of a population-based survey

**DOI:** 10.1186/s40359-017-0174-3

**Published:** 2017-03-06

**Authors:** Johanna M. Doerr, Daniela S. Jopp, Michael Chajewski, Urs M. Nater

**Affiliations:** 1 Clinical Biopsychology, Dept. of Psychology, University of Marburg, Gutenbergstrasse 18, 35032 Marburg, Germany; 2 Dept. of Psychology, Fordham University, Dealy 318, 441 East Fordham Road, Bronx, NY 10458-9993 USA

**Keywords:** Chronic fatigue syndrome, Control beliefs, Personality, Coping

## Abstract

**Background:**

Chronic fatigue syndrome (CFS) represents a unique clinical challenge for patients and health care providers due to unclear etiology and lack of specific treatment. Characteristic patterns of behavior and cognitions might be related to how CFS patients respond to management strategies.

**Methods:**

This study investigates control beliefs in a population-based sample of 113 CFS patients, 264 individuals with insufficient symptoms or fatigue for CFS diagnosis (ISF), and 124 well individuals.

**Results:**

Controlling for personality and coping, individuals with low confidence in their problem-solving capacity were almost 8 times more likely to be classified as ISF and 5 times more likely to be classified as CFS compared to being classified as well. However there was a wide distribution within groups and individuals with “low confidence” scores were found in 31.7% of Well individuals. Individuals with low levels of anxiety and who were more outgoing were less likely to be classified as ISF or CFS.

**Conclusions:**

These findings suggest that fostering control beliefs could be an important focus for developing behavioral management strategies in CFS and other chronic conditions.

## Background

Chronic fatigue syndrome (CFS) is a highly disabling chronic illness with no clear set of pathognomonic clinical signs or diagnostic laboratory markers and no clear pathophysiology [38, 43]. It is defined by debilitating fatigue that is not explained by a medical condition and lasts for at least 6 months and is accompanied by a number of additional symptoms such as post-exertional malaise, unrefreshing sleep, muscle and/or joint pain [18]. Management of CFS aims to relieve symptoms and may involve medication for specific symptoms; some previously published recommendations include cognitive behavior therapy, graded exercise therapy and occupational rehabilitation [6, 10, 37]. Although not universally helpful, cognitive behavioral therapy (CBT) and graded exercise have been shown to result in some reduction (moderate effect sizes) in symptom severity and disability in 33 to 70% of the patients (for an overview see e. g. [9, 25, 26, 32]). The underlying mechanisms, however, remain largely unclear [22].

Psychological factors that may influence response to therapy have received increasing attention. The cognitive-behavioral model of CFS management [40, 44, 49] suggests that pathophysiology, clinical presentation and course of the illness involve a complex interplay of physiologic changes in the body with psychological features, such as patients’ illness beliefs (i.e. their cognitive representation of their illness), personality characteristics, and coping strategies.

It is not clear to what extent these psychological features may be involved in the development of CFS and it is likely that they are not unique to CFS. Some features might be the result of the chronicity and severity of the illness. Research on psychological features suggests that these factors may impact the severity and duration of the illness and influence patients’ ability to manage their illness (for an overview see [15, 28]). For example, Prins and colleagues found that a decrease of fatigue severity was most pronounced in those CFS patients who had higher CFS-specific control beliefs at the beginning of CBT treatment [33]. In addition, there is evidence showing that self-efficacy beliefs (i.e. internal control beliefs and self-concept of competence) are amenable to treatment during a multi-component intervention for CFS patients [19]. Although these preliminary findings are promising, more research about the role of control beliefs in CFS is needed. Specifically, it is important to examine more general control beliefs (as opposed to illness-related control beliefs; [24]) as these might constitute specific risk factors for symptom worsening and might thus be targeted by prevention interventions.

General beliefs about controllability, or ‘control beliefs’ may be of importance in CFS. Individuals differ with respect to how much they feel in charge of their lives (i.e. self-efficacy) and how much they feel dominated by external forces (e.g. by chance or powerful others) [23, 41]. More general control beliefs could therefore be important because they serve as an interpretative framework for individual experiences and might shape how patients respond to being ill. As motivational forces, general control beliefs may determine whether individuals develop certain control beliefs regarding their illness and, in turn, how they take an active role in combating an impairing life situation (i.e. coping strategies). For instance, an individual low on control beliefs may feel powerless when faced with fatigue symptoms as they are unable to see if and what they could do about it. By contrast, an individual with high control beliefs may feel encouraged to seek help and to adhere to treatment. Findings are available from several studies with healthy individuals and individuals suffering from chronic diseases other than CFS. They indicate that believing in being able to control important outcomes and having the abilities to produce those outcomes are crucial for solving everyday challenges [41] and for maintaining good health [4, 39]. Scant data exist concerning control beliefs among people with CFS, mostly from studies with illness-related rather than general control beliefs, and findings are inconsistent. Some studies found lower internal health control in adolescents with CFS [46], but other studies found no difference comparing adults with and without CFS [11] or comparing patients with CFS to other chronic diseases [7]. The relation between control beliefs and adaptation is also unclear. One study has shown that about half of all CFS patients tend to invoke internal causal attributions for their illness, but these were unrelated to adaptation [50]. Instead, external control beliefs, including believing that other people have a primary impact, were linked to higher depression [50].

Empirical evidence further suggests a role of individual characteristics such as specific personality traits. For example, in some patients with CFS, higher scores in neuroticism have been observed [5, 8, 14, 29, 45]. Studies on extraversion are less consistent, reporting that CFS patients have higher [27] or lower [8, 29] scores on this personality trait than healthy controls. These observations could also apply to patients with other chronic illnesses and are unlikely to be specific to CFS.

As mentioned above, control beliefs might affect health by influencing coping behaviors, i.e. the behavior and cognitive appraisal people show to manage their illness [17, 24]. Potentially maladaptive coping styles have previously been associated with CFS [13, 28, 30, 31].

The goal of the present study was to identify psychological factors that may be useful in enhancing the effectiveness of therapeutic interventions for CFS. In the present study we concentrated on three central psychological factors: control beliefs, personality, and coping styles. Extending prior findings from the beneficial role of control beliefs in the general population, we hypothesized that general beliefs about control differ between individuals with CFS and healthy controls. Following the established distinction between more internal and external control beliefs, we hypothesized that individuals with CFS have lower beliefs regarding their control potential and competence and higher beliefs regarding chance and powerful others compared to controls.

We also included a group of individuals who were unwell, but showed insufficient symptoms or fatigue to be diagnosed as CFS (ISF). We did so in order to study whether belief patterns were specific to individuals fulfilling the full diagnosis of CFS, or whether they could also be observed in those who have a subclinical expression of chronic fatigue. We also measured personality traits and coping styles, which represent the psychological aspects most strongly investigated in the CFS context so far. Given that findings suggest that individuals with CFS may be more likely to have specific personality and coping patterns, and given that personality and coping relate to the experience of control, it was our goal to determine whether control beliefs have a significant role when concurrently taking personality and coping into account.

## Methods

### Participants

The study was conducted between September 2004 and July 2005 using a cross-sectional design to address a wide variety of questions about the epidemiology and pathophysiology of CFS. Participants were recruited from metropolitan, urban, and rural populations of Georgia using random digit dialing. A first screening interview screened 19,381 residents between the ages of 18 and 59. Of those, 5,623 completed a detailed telephone interview (covering fatigue status, other CFS-like symptoms, and race). Based on these detailed interviews, participants were pre-screened as CFS, ISF (insufficient symptoms or fatigued), or Well, and invited to a 1-day clinical assessment for further assessment of excluding medical conditions. ISF and Well participants were matched to the CFS-like on geographic stratum, sex, race/ethnicity and age. Final classification was done as follows: CFS cases had to fulfill the 1994 case definition and the recommendations by the International CFS Study Group [35]. Specifically, we used the Medical Outcomes Short-Form Health Survey (SF-36; [48]) to determine functional impairment, the Multidimensional Fatigue Inventory (MFI-20; [42]) for fatigue characteristics and the CDC CFS Symptom Inventory to evaluate occurrence, frequency and severity of other somatic symptoms [47]. Subjects classified as CFS had 4 or more CFS case-defining symptoms lasting 6 months or longer, exceeded the Symptom Inventory cut-off, and met the CFS cut-off on the SF-36 and the MFI-20 [36]. Although fatigue is thought of as the major symptom in CFS, there are other important dimensions of the illness such as impaired memory or concentration, unrefreshing sleep, and bodily pain. For many persons with CFS, these symptoms constitute the primary complaint. Therefore, subjects classified as ISF had to meet at least one, but not all CFS criteria (not limited to fatigue), whereas symptoms were not explained by a medical condition (as with the CFS group). Subjects classified as Well met none of the CFS criteria and were not suffering from a medical condition. The design of the study has been described in detail elsewhere [34]. The study protocol was reviewed and approved by the Institutional Review Board of the Centers for Disease Control (CDC IRB # 4121) and all study participants were consented before study participation. The research was conducted in accordance with the Declaration of Helsinki.

The sample of the current analysis included 501 individuals: 113 were classified as CFS, 264 individuals were classified as not meeting full criteria for CFS but reporting at least one of the CFS defining symptoms (insufficient symptoms or fatigue, termed as ISF), and 124 were classified as Well.

### Materials

This study used a selection of reliable and widely established measures for control beliefs, personality and coping. *Control beliefs* were assessed with the Inventory for the Measurement of Self-efficacy and Externality (I-SEE; [20]). Scales include ‘internal control’ (i.e., beliefs about one’s life being determined by oneself), ‘competence’ (i.e., beliefs about one’s life-management and problem-solving capacity), ‘powerful others’ (i.e., beliefs about other people controlling one’s life), and ‘chance ‘(i.e., beliefs about one’s life being controlled by accidental happenings) (8 items per subscale). Response options were − 3 = strongly disagree to + 3 = strongly agree. Reliability was acceptable to good (internal control: Cronbach’s α = .62; competence: α = .70; powerful others: α = .78, and chance: α = .80). No reliability differences between groups were observed.

### Personality traits

‘Neuroticism’ (i.e. being anxious, moody, or worrisome), ‘extraversion’ (i.e. being outgoing and talkative), ‘openness to experience’ (i.e. displaying intellectual curiosity, preference for variety), ‘agreeableness’ (i.e. being cooperative and considerate), and ‘conscientiousness’ (i.e. being thorough and careful) were assessed with the NEO Five Factor Inventory-NEO-FFI [12]. Cronbach’s αs ranged from .72 (openness) to .89 (neuroticism). No group differences in reliability were found.

### Coping styles

Coping styles were assessed with the Ways of Coping Questionnaire (WCQ; [16]), measuring cognitive and behavioral strategies (66 items). Scales include ‘confrontive coping’ (exemplary item: ‘I stood my ground and fought for what I wanted’) (α = .70), ‘distancing’ (e.g. ‘Tried to forget the whole thing’) (α = .66), ‘self-controlling’ (e.g. ‘I tried to keep my feelings to myself’) (α = .69), ‘seeking social support’ (e.g. ‘I got professional help’) (α = .75), ‘accepting responsibility’ (e.g. ‘Criticized or lectured myself’) (α = .71), ‘escape-avoidance’ (e.g. ‘Took it out on other people’) (α = .73), ‘planful problem solving’ (e.g. ‘I made a plan of action and followed it’) (α = .76), and ‘positive reappraisal’ (e.g. ‘I changed something about myself’) (α = .83). Reliabilities were comparable across groups, except for lower values for ‘distancing’ (.59) and ‘escape-avoidance’ (.56) in the *Well* group.

### Analysis plan

Group differences in mean levels of control beliefs, personality, and coping were tested using ONEWAY ANOVAs with Post-hoc Scheffé tests (two tailed). χ^2^ tests were conducted for sex and race. Being the most appropriate procedure when comparing three groups (three separate logistic regressions would increase the likelihood for Type I error), a multinomial logistic regression analysis was used to examine whether control beliefs were associated with the likelihood of being a member of the CFS, ISF or Well groups. We tested a model including control beliefs, personality traits, and coping styles concurrently, and further added chronological age, sex (men, women) and race (white, non-white). Reported odds ratios are adjusted for all variables in the model. For better interpretability we transformed the continuous data into categorical variables based on the sample’s distribution of each variable. Each predictor (e.g., internal control) had three categories: low (i.e., those 33.3% of the sample low on internal control), medium (those 33.3% with medium internal control), and high (those 33.3% high on internal control beliefs). For this analysis, we excluded one person with CFS and one with ISF based on tests for multivariate outliers (using Mahalanobis Distance). There was no indication for multicollinearity. Type I error rate rejection level for all analyses was set to *p* = .05.

## Results

### Mean level differences in control, personality traits, and coping styles

CFS, ISF, and Well participants did not differ in age, sex, or race (Table 1). Mean levels of internal control and competence beliefs were significantly lower in the CFS compared to the Well group. ISF cases had reduced levels of competence beliefs similar to the CFS group. Their level of internal control was in between the levels of the CFS and the Well group, but there were no significant differences between ISF and CFS. Despite significant differences in mean levels, belief levels varied strongly across individuals. For example, low levels of internal control (defined as being below median) occurred in all three groups (i.e., 64.0% in CFS, 55.3% in ISF, and 41.5% in Well). The same was the case for competence beliefs, showing low beliefs levels in all groups: CFS (66.7%), ISF (60.6%) and the Well (31.7%). Thus, although low belief levels were more frequent in CFS and ISF individuals compared to the Well group, there were also CFS and ISF individuals with medium and high internal control beliefs.

**Table 1:** Sample Characteristics and Central Constructs for CFS, ISF, and Well Group

	Groups									
Variable	CFS (*n* = 113)		ISF (*n* = 264)		Well (*n* = 124)			*p*		
	*n (%)*		*n (%)*		*n (%)*					
*Sex*										
Female	92 (81.4)		201 (76.1)		93 (75)			.44		
Male	21 (18.6)		63 (23.9)		31 (25)					
*Race*										
White	84 (74.3)		196 (74.2)		95 (76.6)			.87		
Non White	29 (25.7)		68 (25.8)		29 (23.4)					
	*M (SD)*	*95% CI*	*M (SD)*	*95% CI*	*M (SD)*	*95% CI*	*F*	*p*	*η* ^*2*^	post-hoc comparison
*Age (years)*										
Mean (*SE*)	44.29 (.95)	42.41–46.17	43.11 (.95)	41.85–44.37	44.52 (.94)	42.66–46.39	1.01	.37	0.00	
Range	18–59		18–59		19–59					
*Control beliefs*										
Internal control	7.19 (.56)	6.07–8.31	8.60 (.39)	7.85–9.36	10.17 (.54)	9.11–11.23	7.00	.004	0.03	C = I; C < W; I = W
Competence	6.17 (.65)	4.88–7.47	7.35 (.44)	6.49–8.22	12.17 (.51)	11.16–13.18	28.56	<.001	0.10	C = I < W
Powerful others	−10.19 (.78)	−11.74– − 8.64	−10.25 (.49)	−11.21– − 9.29	−12.09 (.64)	−13.36– − 10.82	2.63	.28	0.01	
Chance	−10.80 (.77)	−12.33– − 9.27	−11.76 (.50)	−12.75– − 10.77	−12.89 (.68)	−14.23– − 11.55	2.01	.56	0.01	
*Personality*										
Neuroticism	1.98 (.07)	1.84–2.12	1.67 (.04)	1.58–1.75	.92 (.04)	0.83–1.01	84.01	<.001	0.25	C > I > W
Extraversion	2.04 (.05)	1.93–2.15	2.24 (.03)	2.18–2.31	2.66 (.04)	2.57–2.75	41.34	<.001	0.14	C < I < W
Openness	2.27 (.04)	2.19–2.35	2.19 (.03)	2.13–2.26	2.29 (.05)	2.20–2.38	1.87	.16	0.01	
Agreeableness	2.79 (.04)	2.71–2.88	2.79 (.03)	2.74–2.84	3.06 (.03)	2.99–3.13	17.01	<.001	0.06	C = I < W
Conscientiousness	2.71 (.05)	2.61–2.81	2.82 (.03)	2.76–2.88	3.07 (.04)	2.99–3.15	16.56	<.001	0.06	C = I < W
*Coping*										
Confrontive	12.27 (.35)	11.59–12.96	11.05 (.21)	10.63–11.47	9.80 (.26)	9.28–10.32	15.69	<.001	0.06	C = I > W
Distancing	11.58 (.33)	10.92–12.24	11.45 (.21)	11.04–11.87	10.41 (.26)	9.90–10.93	5.00	.056	0.02	
Self-Controlling	16.35 (.39)	15.57–17.12	15.72 (.27)	15.20–16.25	14.64 (.38)	13.89–15.39	4.96	.056	0.02	
Seeking Support	12.81 (.36)	12.11–13.52	12.64 (.26)	12.12–13.16	12.28 (.35)	11.59–12.98	0.54	>1.00	0.00	
Responsibility	7.98 (.30)	7.39–8.57	7.20 (.17)	6.86–7.54	6.23 (.22)	5.80–6.67	11.56	<.001	0.04	C = I; C > W; I = W
Escape-Avoidance	14.25 (.45)	13.35–15.15	13.27 (.26)	12.76–13.77	10.91 (.26)	10.39–11.43	22.04	<.001	0.08	C = I > W
Problem Solving	14.26 (.39)	13.48–15.03	14.18 (.25)	13.68–14.68	13.91 (.41)	13.11–14.71	0.24	>1.00	0.00	
Reappraisal	15.16 (.47)	14.23–16.09	14.97 (.34)	14.30–15.65	14.47 (.46)	13.55–15.39	0.56	>1.00	0.00	

*Note.* Higher scores indicate stronger beliefs, *M* mean, *SD* standard deviation*, 95% CI* 95% confidence intervals for the means, ONEWAY (Analysis of Variance), Kruskal-Wallis for sex and race with *F* and post-hoc comparison (Scheffé) for continuous outcomes (significance level Bonferroni adjusted); Bonferroni-adjusted *p*; effect size *η* ^*2*^ *= between-groups effect/total amount of variance*

Personality traits mean levels differed between CFS, ISF and Well groups (Table 1). Neuroticism scores differed significantly between all groups, and were highest in the CFS and lowest in the Well group. Extraversion scores were lowest for CFS and highest in the Well group. Agreeableness and conscientiousness were lower in CFS and ISF compared to the Well group.

Coping styles also differed between groups (Table 1). Confrontive coping, responsibility taking, and escape-avoidance were higher in CFS than in the Well group.

### Correlational analysis linking control beliefs, personality traits, and coping styles

In line with theoretical expectations, the two scales capturing internal beliefs were positively correlated (internal control, competence: *r* = .44, *p* < .001). The two external belief scales were also positively correlated, but their link was substantially stronger (powerful others, chance: *r* = .63, *p* < .001). Competence was strongly correlated with chance (*r* = −.48) and powerful others (*r* = −.43, *p*s < .001). Internal control was related to chance (*r* = −.12, *p* < .01).

Competence beliefs were negatively related to neuroticism (*r* = −.59), and positively related to extraversion and conscientiousness (*r* = .52, and *r* = .43, *p*s < .001). Internal control had a comparable, but less strong pattern. Powerful others and chance had positive links to neuroticism (*r* = .31, and *r* = .32), and negative links to agreeableness (*r* = −.26, and *r* = −.32), extraversion (*r* = −.17, and *r* = −.24) and conscientiousness (*r* = −.13, *p* < .01, and *r* = −.16, *p*s < .001).

Control beliefs were also significantly correlated with coping styles. However, their correlations were generally lower than those with personality traits. The strongest links existed for escape-avoidance coping, which was negatively correlated with competence (*r* = −.36) and positively correlated with chance beliefs (*r* = .35, *p*s < .001). The other correlations, if significant, ranged between − .10 and .25. Some scales showed no relations to beliefs (e.g., seeking support). Notably, correlation patterns did not differ between CFS, ISF and Well groups.

### Regressions linking control beliefs, personality traits, and coping style to CFS and ISF

Multinomial logistic regression was used to test whether control beliefs were associated with differential classification as CFS or ISF as compared to the Well group. The Deviance test indicated a good model fit. The model had a classification rate of 65%, predicting the classification 38% better than chance (Kappa = .38). The omnibus test revealed effects for competence, neuroticism, extraversion, openness, agreeableness, and confrontive coping. For exact values of the test see Table 2.

**Table 2:** Results of multinomial logistic regression

		Target Group			
Cofactors	Omnibus Test	CFS vs Well		ISF vs Well	
	χ^*2*^	*OR (95% CI)*	*p*	*OR (95% CI)*	*p*
Control beliefs					
Internal control (high = 1)	5.86	––		––	
Medium Internal control		1.62 (0.71–3.72)	.257	0.75 (0.38 – 1.47)	.397
Low Internal control		1.21 (0.49–2.96)	.683	0.66 (0.32 – 1.38)	.273
Competence (high = 1)	17.03**	––		––	
Medium competence		1.21 (0.51–2.83)	.663	1.69 (0.86–3.32)	.129
Low competence		5.91 (1.67–20.96)	.006	8.69 (2.83–26.69)	<.001
Powerful others (high = 1)	1.92	––		––	
Medium Powerful others		1.32 (0.54–3.23)	.545	1.45 (0.69–3.07)	.330
Low Powerful others		1.76 (0.68–4.56)	.247	1.45 (0.65–3.22)	.359
Chance (high = 1)	8.01	––		––	
Medium Chance		1.04 (0.43–2.50)	.938	1.77 (0.85–3.68)	.127
Low Chance		2.15 (0.80–5.80)	.130	2.76 (1.19–6.40)	.018
Personality					
Neuroticism (high = 1)	36.20**	––		––	
Medium Neuroticism		0.28 (0.09–0.85)	.025	0.32 (0.12–0.88)	.028
Low Neuroticism		0.06 (0.02–0.19)	<.001	0.10 (0.03–0.28)	<.001
Extraversion (high = 1)	12.36*	––		––	
Medium Extraversion		3.22 (1.39–7.46)	.007	2.04 (1.05–3.97)	.036
Low Extraversion		3.42 (1.22–9.61)	.019	1.29 (0.56–2.99)	.555
Openness (high = 1)	14.25**	––		––	
Medium Openness		0.80 (0.37–1.77)	.585	0.75 (0.39–1.47)	.403
Low Openness		0.48 (0.21–1.14)	.098	1.34 (0.69–2.62)	.392
Agreeableness (high = 1)	11.89*	––		––	
Medium Agreeableness		0.95 (0.43–2.07)	.893	1.98 (1.07–3.68)	.031
Low Agreeableness		1.95 (0.75–5.08)	.171	3.08 (1.35–7.02)	.008
Conscientiousness (high = 1)	0.85	––		––	
Medium Conscientiousness		0.85 (0.38–1.90)	.683	1.04 (0.55–1.96)	.904
Low Conscientiousness		0.90 (0.36–2.26)	.820	1.22 (0.56–2.64)	.622
Coping					
Confrontive (high = 1)	18.49**	––		––	
Medium Confrontive		0.22 (0.09–0.53)	.001	0.73 (0.36–1.49)	.384
Low Confrontive		0.19 (0.07–0.52)	.001	0.69 (0.30–1.63)	.399
Distancing (high = 1)	3.84				
Medium Distancing		0.90 (0.40–2.02)	.796	0.83 (0.43–1.60)	.571
Low Distancing		0.76 (0.28–2.05)	.579	0.48 (0.21–1.08)	.075
Self-Controlling (high = 1)	3.59	––		––	
Medium Self-Controlling		0.50 (0.21–1.21)	.125	0.55 (0.26–1.16)	.117
Low Self-Controlling		0.45 (0.15–1.38)	.162	0.67 (0.27–1.66)	.382
Seeking Support (high = 1)	1.77	––		––	
Medium Seeking Support		1.36 (0.60–3.12)	.462	0.89 (0.45–1.77)	.993
Low Seeking Support		1.25 (0.43–3.57)	.684	1.00 (0.43– 2.32)	.743
Responsibility (high = 1)	2.68	––		––	
Medium Responsibility		0.70 (0.30–1.65)	.410	1.08 (0.53–2.19)	.832
Low Responsibility		1.00 (0.36–2.82)	.998	0.95 (0.41–2.21)	.902
Escape-Avoidance (high = 1)	2.60	––		––	
Medium Escape-Avoidance		0.92 (0.39–2.16)	.839	0.79 (0.39–1.61)	.517
Low Escape-Avoidance		1.62 (0.56–4.71)	.375	0.86 (0.37–2.01)	.721
Problem Solving (high = 1)	3.07	––		––	
Medium Problem Solving		1.25 (0.56–2.80)	.589	1.10 (0.57–2.13)	.777
Low Problem Solving		1.24 (0.45–3.38)	.681	0.70 (0.31–1.59)	.391
Reappraisal (high = 1)	5.17	––		––	
Medium Reappraisal		0.65 (0.26–1.62)	.357	0.73 (0.35–1.53)	.399
Low Reappraisal		1.11 (0.37–3.38)	.849	1.62 (0.67–3.93)	.283

*Note*. Multinomial Logistic Regression.**p<.010, *p<.050 ORs are adjusted for all other variables in the model. Well = reference group; high = reference category; also included in the analysis: age (covariate), sex (male, female), race (Caucasian, Other). If not otherwise specified, variables were divided into tertiles based on their distribution in this sample (low: 0–33%, medium: 34–66%, high: 67–100% of the sample). Model fit χ2 (df = 74, *n* = 488) = 229.00, *p* < .001, Deviance χ2 (df = 898, *n* = 488) = 765.54, *p* = .999. Omnibus test: dfs for age: 1; sex and race: df = 2, all other variables: df = 4, *Pseudo-R* ^*2*^ (Nagelkerke) = 0.43

**Fig. 1:**
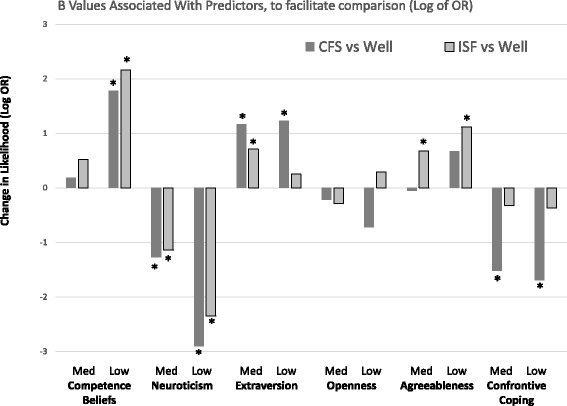
B values (Log of OR) for CFS or ISF (relative to Well individuals) associated with Control Beliefs, Personality Traits and Coping Strategies (significant effects only)

Comparing ISF and Well groups showed that individuals with lower competence beliefs were more likely to belong to the ISF than the Well group. This effect was the strongest in the analysis: When having low competence beliefs, individuals were almost 8 times more likely to be classified as ISF compared to being classified as Well (OR = 8.69, see Table 2 and Fig. 1). Low neuroticism scores were linked to lower odds for being in the ISF group and moderate extraversion scores were associated with higher likelihood for ISF, both relative to higher scores. Further, low agreeableness was related with higher odds for being classified as ISF than Well.

Comparing CFS and Well groups showed that low levels of competence beliefs were related to a higher likelihood of being classified as CFS: Individuals with low competence beliefs were 5 times more likely to be classified as CFS compared to being classified as Well (OR = 5.91, see Table 2).

Notably, personality traits played a somewhat more important role in this CFS vs. Well comparison: Besides a comparable effect of neuroticism, with subjects scoring low on this scale having a lower likelihood to be in the CFS group, individuals with low scores in openness also had a reduced risk relative to highly open individuals to belong to the CFS group. Having low or moderate scores in extraversion, by contrast, was related to a higher chance of being classified as CFS. Individuals low on confrontive coping were less likely to be in the CFS group than individuals with high scores.

Finally, the comparison between CFS and ISF (Fig. 1) suggests that although low competence beliefs increased the likelihood for CFS and ISF classification, odds were much higher for ISF. Low extraversion was related to higher odds for CFS than ISF classification. Lower openness and lower confrontive coping were also related to a reduced likelihood for CFS relative to ISF.

## Discussion

Our study suggests that general control beliefs should be studied in relation to how CFS patients respond to therapy. Individuals classified as CFS or ISF had less confidence in their ability to realize goals and to solve everyday problems compared to well individuals. More specifically, CFS individuals felt on average less in control than did ISF individuals, who in turn displayed lower levels of competence beliefs than well individuals. However there was considerable variation in the CFS group, with only about two thirds reporting low competence control. Although the number of people with at risk competence beliefs was higher in the CFS than in the ISF and the Well groups, it clearly shows that not all individuals with CFS had lower general competence beliefs. Prior findings underscored the relevance of specific fatigue-related beliefs [28]. For example, White and colleagues [50] found that external causal attributions for CFS were related to poorer psychological adjustment among CFS patients. It is reasonable that general control beliefs foster fatigue-specific control beliefs (or illness beliefs). However, this association should be addressed in greater detail in future studies. The variance in control beliefs underscores the complexity of CFS and the need to use more comprehensive models which include cognitive-behavioral as well as biological variables.

This cross-sectional data does not indicate causality, and it is noteworthy that the levels of beliefs varied strongly across individuals. Possibly, control beliefs represent one of the factors that may help identify which individuals with CFS are likely to benefit from a cognitive behavioral intervention. One study indeed found that fatigue severity decrease was most pronounced in those CFS patients who had higher CFS specific control beliefs at the beginning of CBT treatment [33]. Intervention studies are needed to test directionality of changes in control beliefs and changes in symptoms.

Control beliefs, neuroticism, openness, extraversion, agreeableness, and confrontive coping were associated with classifying individuals as CFS or ISF. Control beliefs, particularly low confidence in their competence to accomplish goals and solve problems, were associated with CFS and ISF. Interestingly, there were no differences between groups in externality, and internal control beliefs did not turn out to be a relevant predictor in the regression model with all other variables controlled. Our study findings suggest that control beliefs are prominent features in ISF and CFS, possibly at least as important as personality traits and coping styles as had been found in prior research [29–31]. Our current study tested the value of control beliefs, personality traits, and coping strategies concurrently and support the notion of a complex model for the clinical approach to CFS. To our knowledge, this is also one of the first studies on general control beliefs in the context of CFS.

The importance of general control beliefs in the CFS context may offer an additional route for cognitive-behavioural therapy. Our study indicates that more global (i.e., health-unspecific) perceptions of control may also be relevant, representing an underlying tendency to experience one’s life that could be directly addressed in behavioral interventions, such as CBT.

Self-efficacy which is defined as “people’s beliefs about their capability to exercise control over events that affect their lives” ([1], p. 1175) can be seen as a “function” of internal control beliefs and competence beliefs. Notably, self-efficacy beliefs were also found to be amenable by treatment during a multi-component intervention for CFS patients [19]. Thus, addressing competence beliefs in interventions might be worthwhile. Particularly, evidence-based mechanisms enhancing self-efficacy such as self-management (e.g., set realistic goals, initiate and monitor activities, reflection on past successes [2] and activity management (e.g., combination of pacing and rest, gradual increase of activity; [19]) could be helpful for patients with low levels of competence beliefs.

Another noteworthy finding is the difference between CFS and ISF. While CFS and ISF patients showed the same association pattern with respect to control beliefs compared to well controls, personality traits and coping styles were only associated with CFS, but not with ISF. If ISF and CFS are seen on a continuum, with ISF being a group comprised of individuals not yet manifesting the full symptomology of CFS, one might hypothesize that control beliefs may be associated with the development of ISF into CFS. Until now, cognitive behavioral models of CFS have focused on personality traits as a predisposing factor and illness beliefs and coping styles as maintaining factors [28, 44]. Our findings suggest that including general control beliefs in these models might be an important theoretical addition. As explained above, one might suspect competence beliefs could be a risk factor for transitioning from ISF to CFS. However, this question can only be answered using a longitudinal design.

Others have noted the difficulties in treating CFS patients [3]. As shown by prior work and our current study, individuals with CFS score higher on neuroticism, which makes them more prone to perceive experiences as stressful and to respond to difficult situations with both anxiety and depression. Individuals scoring high on neuroticism are also likely to display potentially maladaptive behavior (e.g., avoidance) and show high levels of resistance to psychological treatment. The negative effects of neuroticism may be enhanced if combined with the belief of not being able to handle stressful situations successfully, i.e. poor competence beliefs. These factors may have developed due to the chronicity and severity of the illness, and are unlikely to be specific to CFS, but could help improve therapeutic interventions.

Considering the symptom overlap between CFS and other syndromes that are associated with fatigue and/or pain, it would be of great interest to study the role of control beliefs in conditions such as fibromyalgia or irritable bowel syndrome (i.e. chronic overlapping pain conditions); it is likely that low control beliefs are not specific for CFS, but might be a general factor relevant for other syndromes.

Study limitations include the cross-sectional nature of the data, which does not allow causal inference. Another limitation is the lack of using recently advocated guidelines of CFS definition by the Institute of Medicine [21]. The IOM guidelines stipulate that the post-exertional malaise is a core feature of CFS. In the 1994 case definition post-exertional malaise (PEM) is a case-defining symptom, but not required for diagnosis. While 89% of participants classified as CFS in this study did endorse PEM, findings could be different if analysis was restricted to this subgroup. Finally, although we examined a population-based sample, in which we included participants between the ages of 18 and 59 from metropolitan, urban, and rural populations of Georgia, our findings are not representative for younger or older populations or populations from other cultural backgrounds. Longitudinal studies comparing patients with CFS and patients with other chronic diseases with respect to control beliefs and their response to therapeutic interventions are needed.

## Conclusions

Our findings highlight the so far overlooked role of general control beliefs of adults in the context of CFS. They suggest that addressing CFS and ISF patients’ general beliefs may increase the likelihood of successful therapeutic interventions. Teaching individuals to use specific coping strategies may be improved if competence beliefs are addressed concurrently, since poor competence beliefs are likely to hinder the effective application of coping strategies. Thus, strengthening the CFS and ISF patients’ beliefs in their competence to reach their goals under normal and difficult circumstances may enhance the success of therapeutic interventions.

## Abbreviations

CBT: Cognitive behavioural therapy
CFS: Chronic fatigue syndrome
I-SEE: Inventory for the measurement of self-efficacy and externality
ISF: Insufficient symptoms or insufficient fatigue to be classified as CFS
NEO-FFI: NEO Five factor inventory
WCQ: Ways of coping questionnaire

## References

[1] Bandura A (1989). Human agency in social cognitive theory. Am Psychol.

[2] Bazelmans E, Prins J, Bleijenberg G (2006). Cognitive Behavior Therapy for Relatively Active and for Passive Chronic Fatigue Syndrome Patients. Cogn Behav Pract.

[3] Bazelmans E, Prins JB, Hoogveld S, Bleijenberg G (2004). Manual-based cognitive behaviour therapy for chronic fatigue syndrome: therapists’ adherence and perceptions. Cogn Behav Ther.

[4] Bisschop MI, Kriegsman DM, Beekman AT, Deeg DJ (2004). Chronic diseases and depression: the modifying role of psychosocial resources. Soc Sci Med.

[5] Blakely AA, Howard RC, Sosich RM, Murdoch JC, Menkes DB, Spears GF (1991). Psychiatric symptoms, personality and ways of coping in chronic fatigue syndrome. Psychol Med.

[6] Boneva RS, Decker MJ, Maloney EM, Lin JM, Jones JF, Helgason HG, Reeves WC (2007). Higher heart rate and reduced heart rate variability persist during sleep in chronic fatigue syndrome: a population-based study. Auton Neurosci.

[7] Buchwald D, Garrity D (1994). Comparison of patients with chronic fatigue syndrome, fibromyalgia, and multiple chemical sensitivities. Arch Intern Med.

[8] Buckley L, MacHale SM, Cavanagh JT, Sharpe M, Deary IJ, Lawrie SM (1999). Personality dimensions in chronic fatigue syndrome and depression. J Psychosom Res.

[9] Castell BD, Kazantzis N, Moss-Morris R (2011). Cognitive Behavioral Therapy and Graded Exercise in Chronic Fatigue Syndrome: A Meta-Analysis. Clin Psychol Sci Pract.

[10] Chambers D, Bagnall AM, Hempel S, Forbes C (2006). Interventions for the treatment, management and rehabilitation of patients with chronic fatigue syndrome/myalgic encephalomyelitis: an updated systematic review. J R Soc Med.

[11] Cope H, Mann A, Pelosi A, David A (1996). Psychosocial risk factors for chronic fatigue and chronic fatigue syndrome following presumed viral illness: a case–control study. Psychol Med.

[12] Costa PT, McCrae RR (1992). NEO PI-R professional manual.

[13] Creswell C, Chalder T (2001). Defensive coping styles in chronic fatigue syndrome. J Psychosom Res.

[14] Fiedler N, Lange G, Tiersky L, DeLuca J, Policastro T, Kelly-McNeil K, Natelson B (2000). Stressors, personality traits, and coping of Gulf War veterans with chronic fatigue. J Psychosom Res.

[15] Fischer S, Nater UM, Hudson C (2014). Illness Perception and Management of Chronic Fatigue Syndrome. Chronic Fatigue Syndrome: Risk Factors, Management and Impacts on Daily Life.

[16] Folkman S, Lazarus R (1988). Manual for the Ways of Coping Questionnaire.

[17] Folkman S, Moskowitz JT (2004). Coping: Pitfalls and Promise. Annu Rev Psychol.

[18] Fukuda K, Straus SE, Hickie I, Sharpe MC, Dobbins JG, Komaroff A (1994). The chronic fatigue syndrome: a comprehensive approach to its definition and study. International Chronic Fatigue Syndrome Study Group. Ann Intern Med.

[19] Goudsmit EM, Ho-Yen DO, Dancey CP (2009). Learning to cope with chronic illness. Efficacy of a multi-component treatment for people with chronic fatigue syndrome. Patient Educ Couns.

[20] Greve W, Anderson A, Krampen G (2001). Self-efficacy and externality in adolescence: Theoretical conceptions and measurement in New Zealand and German secondary school students. Identity.

[21] Institute of Medicine, Board on the Health of Select Populations (2015). Beyond Myalgic Encephalomyelitis/Chronic Fatigue Syndrome: Redefining an Illness.

[22] Knoop H, Bleijenberg G, Gielissen MF, Van der Meer JW, White PD (2007). Is a full recovery possible after cognitive behavioural therapy for chronic fatigue syndrome?. Psychother Psychosom.

[23] Levenson H, Lefcourt HM (1981). Differentiating among internality. powerful others, and chance. Research within the locus of control construct.

[24] Leventhal H, Leventhal EA, Contrada R (1998). Self-regulation, health, and behavior: a perceptual-cognitive approach. Psychol Health.

[25] Malouff, J. M., Thorsteinsson, E. B., Rooke, S. E., Bhullar, N., & Schutte, N. S. (2007). Efficacy of cognitive behavioral therapy for chronic fatigue syndrome: A meta-analysis. *Clinical Psychology Review*. doi: 10.1016/j.cpr.2007.10.004. 10.1016/j.cpr.2007.10.004 18060672

[26] Marques MM, De Gucht V, Gouveia MJ, Leal I, Maes S (2015). Differential effects of behavioral interventions with a graded physical activity component in patients suffering from Chronic Fatigue (Syndrome): An updated systematic review and meta-analysis. Clin Psychol Rev.

[27] Masuda A, Munemoto T, Yamanaka T, Takei M, Tei C (2002). Psychosocial characteristics and immunological functions in patients with postinfectious chronic fatigue syndrome and noninfectious chronic fatigue syndrome. J Behav Med.

[28] Moss-Morris R (2005). Symptom perceptions, illness beliefs and coping in chronic fatigue syndrome. J Ment Health.

[29] Nater UM, Jones JF, Lin JM, Maloney E, Reeves WC, Heim C (2010). Personality features and personality disorders in chronic fatigue syndrome: a population-based study. Psychother Psychosom.

[30] Nater UM, Maloney E, Lin JM, Heim C, Reeves WC (2012). Coping styles in chronic fatigue syndrome: findings from a population-based study. Psychother Psychosom.

[31] Nater UM, Wagner D, Solomon L, Jones JF, Unger ER, Papanicolaou DA, Heim C (2006). Coping styles in people with chronic fatigue syndrome identified from the general population of Wichita, KS. J Psychosom Res.

[32] Price, J. R., Mitchell, E., Tidy, E., & Hunot, V. (2008). Cognitive behavior therapy for chronic fatigue syndrome in adults (Review). *Cochrane Database Syst Rev, 3*. doi: 0.1002/14651858.CD001027.pub2. 10.1002/14651858.CD001027.pub2 PMC7028002 18646067

[33] Prins JB, Bleijenberg G, Bazelmans E, Elving LD, De Boo TM, Severens JL, Van der Meer JW (2001). Cognitive behaviour therapy for chronic fatigue syndrome: a multicentre randomised controlled trial. Lancet.

[34] Reeves, W. C., Jones, J. F., Maloney, E., Heim, C., Hoaglin, D. C., Boneva, R. S.,…Devlin, R. (2007). Prevalence of chronic fatigue syndrome in metropolitan, urban, and rural Georgia. *Popul Health Metr, 5*, 5. doi: 10.1186/1478-7954-5-5 10.1186/1478-7954-5-5 PMC1904178 17559660

[35] Reeves WC, Lloyd A, Vernon SD, Klimas N, Jason LA, Bleijenberg G, Unger ER (2003). Identification of ambiguities in the 1994 chronic fatigue syndrome research case definition and recommendations for resolution. BMC Health Serv Res.

[36] Reeves WC, Wagner D, Nisenbaum R, Jones JF, Gurbaxani B, Solomon L, Heim C (2005). Chronic fatigue syndrome--a clinically empirical approach to its definition and study. BMC Med.

[37] Reid S, Chalder T, Cleare A, Hotopf M, Wessely S (2000). Chronic fatigue syndrome. BMJ.

[38] Reynolds KJ, Vernon SD, Bouchery E, Reeves WC (2004). The economic impact of chronic fatigue syndrome. Cost Eff Resource Allocation.

[39] Rodin J (1986). Aging and health: effects of the sense of control. Science.

[40] Sharpe M (1998). Cognitive behavior therapy for chronic fatigue syndrome: efficacy and implications. Am J Med.

[41] Skinner EA (1996). A guide to constructs of control. J Pers Soc Psychol.

[42] Smets EM, Garssen B, Bonke B, De Haes JC (1995). The Multidimensional Fatigue Inventory (MFI) psychometric qualities of an instrument to assess fatigue. J Psychosom Res.

[43] Solomon L, Nisenbaum R, Reyes M, Papanicolaou DA, Reeves WC (2003). Functional status of persons with chronic fatigue syndrome in the Wichita, Kansas, population. Health Qual Life Outcomes.

[44] Surawy C, Hackmann A, Hawton K, Sharpe M (1995). Chronic fatigue syndrome: a cognitive approach. Behav Res Ther.

[45] Taillefer SS, Kirmayer LJ, Robbins JM, Lasry JC (2003). Correlates of illness worry in chronic fatigue syndrome. J Psychosom Res.

[46] van de Putte EM, Engelbert RH, Kuis W, Sinnema G, Kimpen JL, Uiterwaal CS (2005). Chronic fatigue syndrome and health control in adolescents and parents. Arch Dis Child.

[47] Wagner D, Nisenbaum R, Heim C, Jones JF, Unger ER, Reeves WC (2005). Psychometric properties of the CDC Symptom Inventory for assessment of chronic fatigue syndrome. Popul Health Metrics.

[48] Ware JE, Sherbourne CD (1992). The MOS 36-item short-form health survey (SF-36). I. Conceptual framework and item selection. Med Care.

[49] Wessely S, Butler S, Chalder T, David A, Jenkins R, Mowbray JF (1991). The cognitive behavioural managment of the post-viral fatigue syndrome. Post-viral fatigue syndrome.

[50] White K, Lehman DR, Hemphill KJ, Mandel DR, Lehman AM (2006). Causal Attributions, Perceived Control, and Psychological Adjustment: A Study of Chronic Fatigue Syndrome. J Appl Soc Psychol.

